# Antifungal Effect of All-*trans* Retinoic Acid against Aspergillus fumigatus *In Vitro* and in a Pulmonary Aspergillosis *In Vivo* Model

**DOI:** 10.1128/AAC.01874-20

**Published:** 2021-02-17

**Authors:** Elena Campione, Roberta Gaziano, Elena Doldo, Daniele Marino, Mattia Falconi, Federico Iacovelli, Daniela Tagliaferri, Lucrezia Pacello, Luca Bianchi, Caterina Lanna, Luigi Aurisicchio, Federica Centofanti, Paolo Di Francesco, Ilaria Del Principe, Francesca Del Bufalo, Franco Locatelli, Enrico Salvatore Pistoia, Emanuele Marra, Augusto Orlandi

**Affiliations:** a Dermatologic Unit, Department of Systems Medicine, University of Rome Tor Vergata, Rome, Italy; b Department of Experimental Medicine, University of Rome Tor Vergata, Rome, Italy; c Anatomic Pathology, Department of Biomedicine and Prevention, University of Rome Tor Vergata, Rome, Italy; d Department of Biology, University of Rome Tor Vergata, Rome, Italy; e Takis S.r.l., Rome, Italy; f Department of Haematology, Oncology and Stem Cell Transplantation, Bambino Gesù Children’s Hospital (IRCSS), Rome, Italy; g Department of Haematology, University of Rome Tor Vergata, Rome, Italy

**Keywords:** *trans*-retinoic acid, *Aspergillus*, invasive pulmonary aspergillosis, ATRA, aspergillosis, pneumonia

## Abstract

Aspergillus fumigatus is the most common opportunistic fungal pathogen and causes invasive pulmonary aspergillosis (IPA), with high mortality among immunosuppressed patients. The fungistatic activity of all-*trans* retinoic acid (ATRA) has been recently described *in vitro*. We evaluated the efficacy of ATRA *in vivo* and its potential synergistic interaction with other antifungal drugs. A rat model of IPA and *in vitro* experiments were performed to assess the efficacy of ATRA against *Aspergillus* in association with classical antifungal drugs and *in silico* studies used to clarify its mechanism of action. ATRA (0.5 and 1 mM) displayed a strong fungistatic activity in *Aspergillus* cultures, while at lower concentrations, synergistically potentiated fungistatic efficacy of subinhibitory concentration of amphotericin B (AmB) and posaconazole (POS). ATRA also enhanced macrophagic phagocytosis of conidia. In a rat model of IPA, ATRA reduced mortality similarly to posaconazole. Fungistatic efficacy of ATRA alone and synergistically with other antifungal drugs was documented *in vitro*, likely by inhibiting fungal heat shock protein 90 (*Hsp90*) expression and Hsp90-related genes. ATRA treatment reduced mortality in a model of IPA *in vivo*. Those findings suggest ATRA as a suitable fungistatic agent that can also reduce dosage and adverse reactions of classical antifungal drugs and add to the development of new therapeutic strategies against IPA and systemic fungal infections.

## Abstract (precis)

Aspergillus fumigatus is the most common opportunistic fungal pathogen and causes invasive pulmonary aspergillosis (IPA), with high mortality among immunosuppressed patients. The fungistatic activity of all-*trans* retinoic acid (ATRA) has been recently described *in vitro*.

## INTRODUCTION

Invasive fungal infection (IFI) is a critical complication in hematologic and neoplastic patients, leading to severe morbidity and mortality (1). IFIs are estimated to be responsible for approximately 1.5 to 2.0 million deaths every year (2). Over the last decades, the incidence of IFIs has increased due to the concurrent growth of immunocompromised patients, the broad use of antibiotics and chemotherapies, bone marrow and organ transplants, and corticosteroids (2). Aspergillus fumigatus is among the most invasive opportunistic pathogens in immunocompromised patients, and the incidence of *Aspergillus*-related IFIs is progressively growing (3). The genus *Aspergillus* includes 4 subgenera (*Aspergillus*, *Circumdati*, *Fumigati*, and *Nidulantes*) and 20 sections, including 339 recognized species (4). Nevertheless, only a few well-known species (including Aspergillus fumigatus and Aspergillus flavus) are considered important opportunistic pathogens in both humans and animals (5). In humans, Aspergillus fumigatus is the most common and life-threatening airborne opportunistic fungal pathogen, with particular significance among immunosuppressed hosts (5). Aspergillus fumigatus causes invasive pulmonary aspergillosis (IPA), which is associated with severe morbidity and mortality (5). Despite the high IPA-related mortality, the most used three classes of antifungal drugs (polyenes, azoles, and echinocandins) display limited clinical efficacy and are often unsuccessful, despite the noteworthy side effects associated with their long-term use, the critical pharmacokinetic profile, and, lastly, the drug-drug interactions (6). An important challenge for the future is to discover novel drugs that can also interact with specific fungal targets or modify the existing ones by optimizing their antifungal action by reducing dosages and consequently side effects.

In the last decades, the role of vitamin A in the regulation of the immune response has been confirmed by several studies, and its deficiency is associated with increased susceptibility to severe infectious diseases (7). Vitamin A is a nutrient obtained through the diet either as provitamin A (carotenoids) or as preformed vitamin A (retinol and retinyl esters), and liver dehydrogenases convert retinol into retinoic acid, its biologically active metabolite (7). Retinoids are key modulators of cell growth, differentiation, and apoptosis through their receptor-regulated signaling pathways (8). In particular, retinoic acid controls the normal immune system development as a modulator of both innate and adaptive immune responses (9). We recently documented that the topical use of the retinoid derivative tazarotene favors rapid healing of fungal nail infections (10); moreover, all-*trans* retinoic acid (ATRA) demonstrated fungistatic activity *in vitro* (11). Based on this evidence, we sought to evaluate ATRA synergistic interaction with conventional antifungal drugs such as amphotericin B and posaconazole in *in vitro* studies and its therapeutic efficacy in a preclinical model of IPA.

## RESULTS

### ATRA displays fungistatic activity on Aspergillus fumigatus germination *in vitro*.

**FIG 1:**
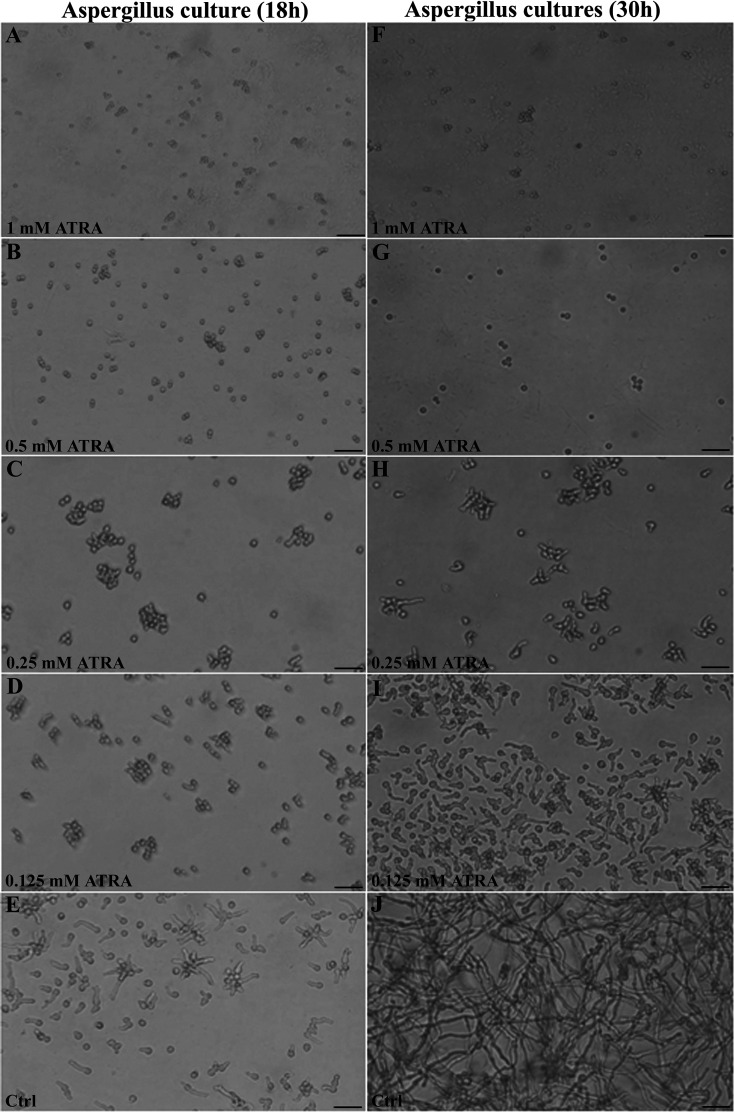
ATRA inhibits germination of Aspergillus fumigatus conidia. Representative images of the effect of different ATRA concentrations on Aspergillus fumigatus conidia germination. The conidia germination was followed using an optical microscope with a ×40 magnification objective lens. Microscopic images were recorded 18 and 30 h after the treatments. One of three representative experiments is shown. Bars indicate 50 µm.

At 0.5 to 1 mM concentration, ATRA exerted a strong inhibitory effect on swelling and germination of *Aspergillus* conidia *in vitro* (Fig. 1A and B) as previously reported (11). After 18 h of treatment with lower ATRA concentrations (0.25 and 0.125 mM), *Aspergillus* conidia appeared swollen (Fig. 1C and D) and similar to control, but without the development of short germ tubes typical of control cultures (Fig. 1E). The inhibitory effect on conidial germination was maintained until 30 h (Fig. 1F and G). At that time, ATRA at concentrations lower than 0.5 mM did not interfere with *Aspergillus* conidia germination, although the development of germ tubes appeared delayed (Fig. 1H and I). Only control *Aspergillus* cultures (Fig. 1L) displayed elongated hyphal structures. The fungistatic effect of ATRA was reversible since conidia were able to germinate and to grow in the hyphal form 7 days after ATRA removal (data not shown).

### ATRA exerts a synergistic fungistatic activity with amphotericin B and posaconazole *in vitro*.

**FIG 2:**
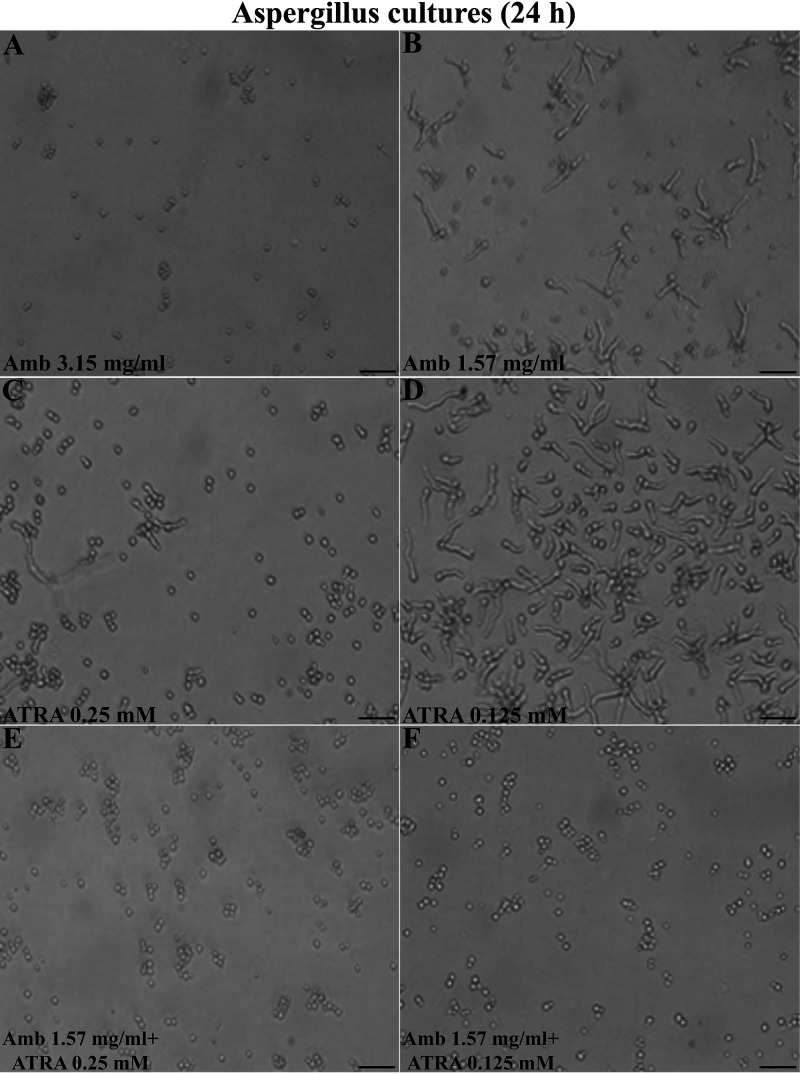
Synergic effect of ATRA and amphotericin B on Aspergillus fumigatus conidia germination. (A) Representative images of the effect of AmB and ATRA alone and in combination on Aspergillus fumigatus conidia germination, followed using an optical microscope with a ×40 magnification objectives lens. Microscopic images were recorded 30 h after the treatments. One of three representative experiments is shown. Bars indicate 50 µm.

In order to investigate the synergistic fungistatic activity of ATRA with standard antifungal drugs, we determined a subinhibitory concentration of amphotericin B (AmB). As shown in Fig. 2A, 3.15 µg/ml was the minimal AmB concentration capable of inhibiting *Aspergillus* conidia germination, while at 1.57 µg/ml concentration, AmB did not interfere with germination (Fig. 2B), and only a delayed hyphal growth was observed. Interestingly, the association of low and noneffective doses of ATRA (0.25 or 0.125 mM; Fig. 2C and D) with that subinhibitory dose of AmB (1.57 µg/ml) displayed a synergistic efficacy against *Aspergillus* conidial swelling and germination after 24 h (Fig. 2E and F), To confirm the synergism of ATRA in combination with AmB or posaconazole (POS), currently used as primary therapy against aspergillosis, antifungal susceptibility tests were also performed *in vitro*. As shown in Table 1, the lowest concentration of ATRA that caused prominent growth inhibition (MIC ≥ 50) of the fungus was 75 µg/ml (0.25 mM), while MIC values of AmB and POS were 3.15 µg/ml and 0.0018 µg/ml, respectively. Of note, both the antifungal drugs showed a synergistic inhibitory effect toward A. fumigatus in combination with a low ineffective dose of 35 µg/ml (0.125 mM) of ATRA. Notably, this synergistic interaction was more remarkable with POS. Indeed, the MIC values of AmB and POS combined with ATRA were one dilution and three dilutions lower, respectively, than the MICs of the drugs alone (MIC of AmB plus ATRA, 1.5 µg/ml [versus MIC of AmB alone, 3.15 µg/ml]; MIC of POS plus ATRA, 0.00022 µg/ml [versus MIC of POS alone, 0.0018 µg/ml]). These results suggest a promising interaction *in vivo* and the therapeutic possibility of reducing classical antifungal drug dosage.

**TABLE 1:** Activity of ATRA alone and combined with amphotericin B or posaconazole on A. fumigatus growth^*a*^

Substance	MIC (µg/ml)
ATRA	75
AmB	3.15
POS	0.0018
ATRA plus AmB	1.57
ATRA plus POS	0.00022

a The MIC is the lowest concentration of ATRA, AmB, or POS alone or in combination at which a prominent decrease in turbidity is observed compared with the drug-free control (≥50% growth inhibition) after 24 h of incubation. In the combination antifungal treatments, ATRA was used at a suboptimal dose of 37.5 µg/ml.

### ATRA reduced mortality in a rat model of invasive pulmonary aspergillosis.

**FIG 3:**
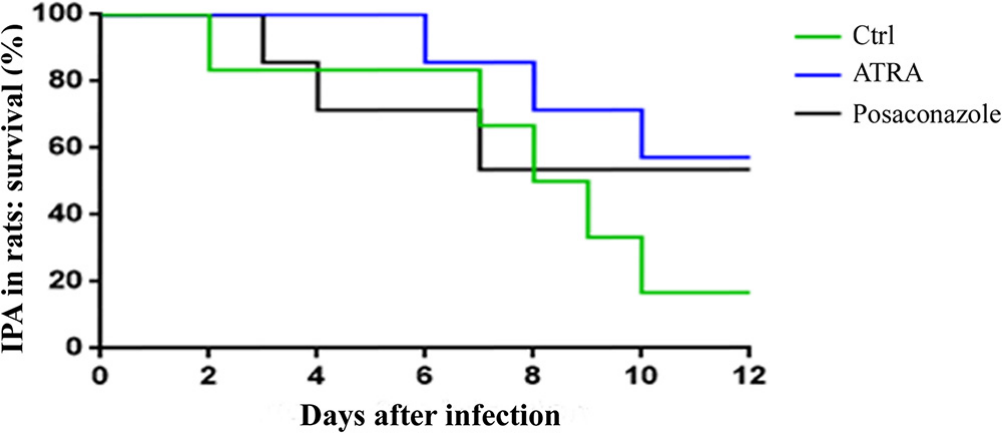
ATRA and posaconazole show a similar efficacy in a rat model of invasive pulmonary aspergillosis. Kaplan-Meier analysis of overall survival of 10 rats/group treated with ATRA (2 mg/kg i.p.), posaconazole (4 mg/kg *per os*), or vehicle (control group). Statistical significance of ATRA and posaconazole treatments versus control group was evaluated by log-rank test analysis; *, *P = *0.05.

We investigated the efficacy of ATRA in a rat model of IPA, also in comparison with the antifungal drug posaconazole, commonly used in clinical practice against IPA. Thirty rats were divided into three experimental groups (*n* = 10), including control, ATRA treated, and posaconazole treated. ATRA dosage and administration protocol (2 mg/kg intraperitoneally), starting 8 days before the infection, were established in a preliminary study (data not shown). Posaconazole treatment (4 mg/kg *per os*) started concurrently with the infection (12). As shown in Fig. 3, both drugs impacted IPA progression compared to the control group. In particular, 100% of ATRA-treated rats were alive 6 days after the infection. During the second week, ATRA and posaconazole displayed a very similar effect on survival, with 60% of rats alive at the end of the study (*P* < 0.05 versus control). Those *in vivo* findings further supported ATRA efficacy as promising for IPA therapy.

### ATRA enhances macrophage phagocytosis of *Aspergillus* conidia *in vitro*.

**FIG 4:**
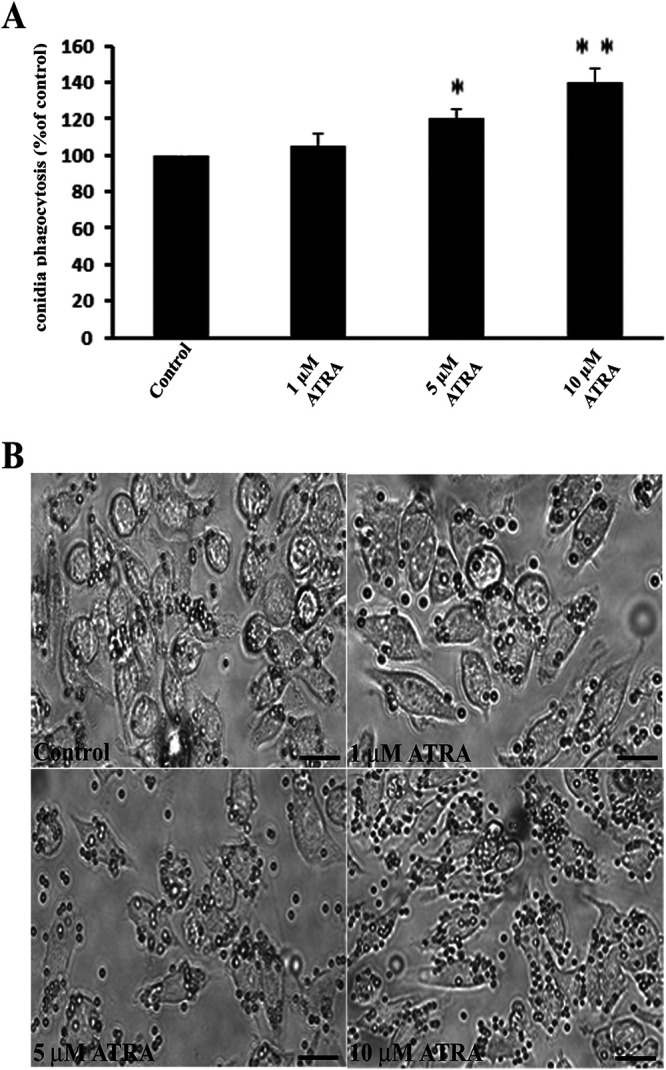
Effect of ATRA on macrophage phagocytosis of Aspergillus fumigatus conidia *in vitro*. (A) Representative image of bar graph showing an increased phagocytosis of Aspergillus fumigatus conidia by human macrophage cell line U-937 incubated overnight with DMSO (control) or different concentrations of ATRA and exposed to unopsonized *Aspergillus* conidia., Data collected from three independent experiments and expressed as percentage of control. Student's *t* test was used to determine the significance of values in experimental groups and defined as *P < *0.05. *, *P < *0.05; **, *P *< 0.01. (B) Representative image of macrophage phagocytosis also performed in 24-well plates. All images of the cell culture plates were recorded at the end of the incubation period using an optical microscope with a ×40 magnification objective lens. One of three representative experiments was shown. Bars indicate 50 µm.

Macrophages play a major role in controlling *Aspergillus* infection (13). In the present study, we also evaluated the capability of ATRA of modulating macrophage phagocytic activity *in vitro*. As reported in Fig. 4A, ATRA significantly increased conidia phagocytosis by U-937 human macrophages compared to control (at 5 or 10 µM, around 20 and 40%, respectively). Image analysis of macrophage phagocytosis (Fig. 4B) confirmed the increased phagocytosis and adherent macrophage-conidia complex after ATRA treatment (5 and 10 µM). Those data suggested ATRA improves macrophage phagocytosis that likely contributes to antifungal activity against IPA *in vivo*.

### ATRA inhibits *Aspergillus* Hsp90 expression and Hsp90-related genes *in vitro*.

**FIG 5:**
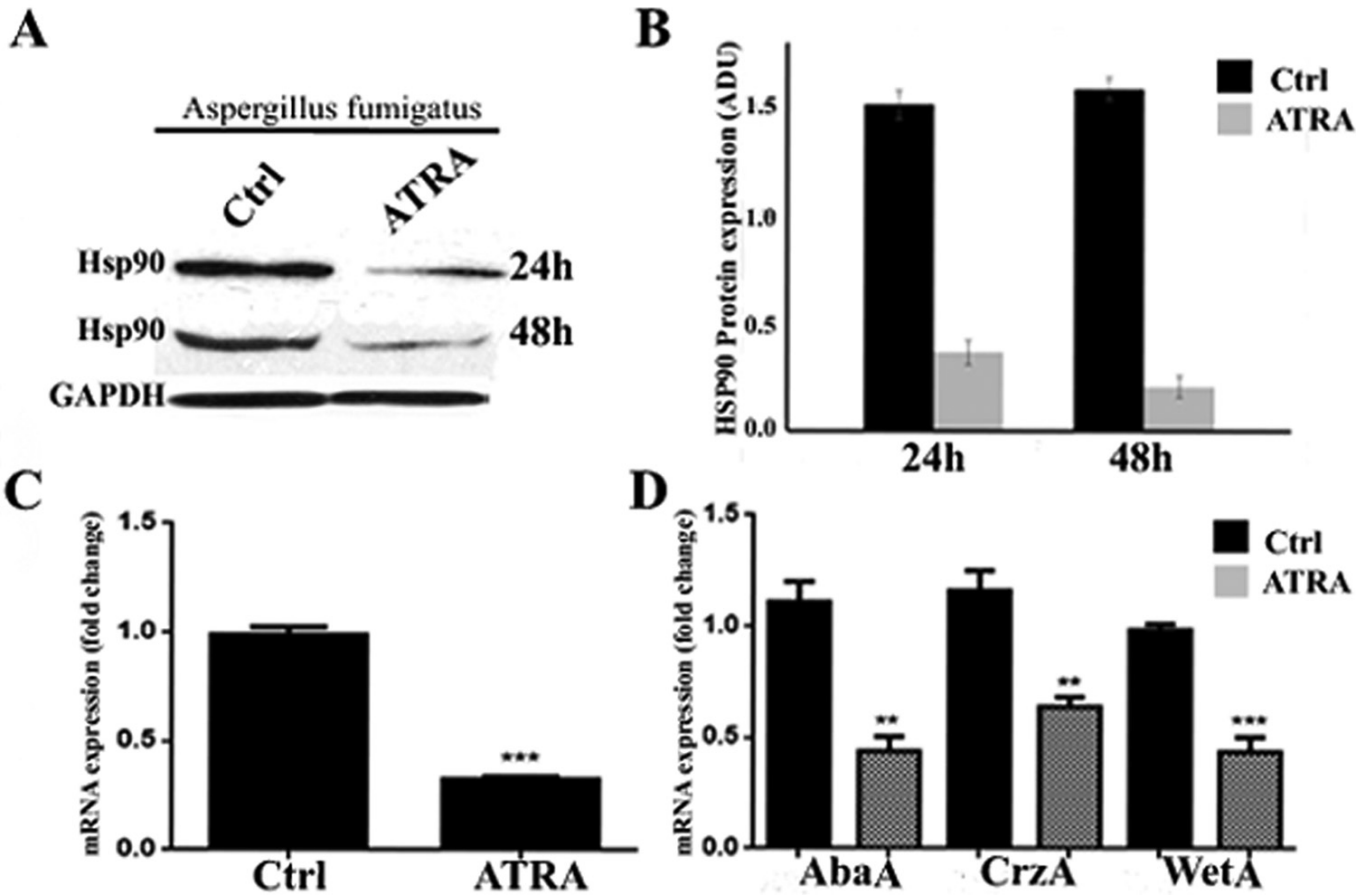
ATRA inhibits the heat shock Hsp90 protein and mRNA expression of *Aspergillus*. Representative blots (A) and bar graphs (B) of densitometric evaluation of Hsp90 protein expression on *Aspergillus* conidia treated with ATRA. *Aspergillus* conidia were cultured in L15 with 2% FCS at 37°C for 24 h in the presence of 1 mM ATRA. The average and SEM of triplicate experiments are shown. Significant changes are reported as *P < *0.001 (***) (Student's *t* test). (C) Hsp90 mRNA expression level assessed by qRT-PCR and normalized to untreated *Aspergillus* conidia. (D). *CrzA*, *WetA*, and *AbaA* mRNA levels assessed by qRT-PCR and normalized to untreated *Aspergillus* conidia. The expression ratios were normalized to elongation factor 1α expression and were calculated according to the threshold cycle (ΔΔ*C_T_*) method. All experiments were performed in triplicate, and data were presented as the mean ± SEM. **, *P < *0.01; ***, *P < *0.001; Student's *t* test.

To confirm whether ATRA is an inhibitor of heat shock protein 90 (*Hsp90*), *Aspergillus* conidia were cultured in ATRA-supplemented medium for 24 h. Immunoblot analysis showed that after 24 and 48 h of ATRA (1 mM) treatment, Hsp90 protein expression was almost suppressed (Fig. 5A and B). In the presence of the reduced density and growth rate of *Aspergillus* conidia, quantitative real-time reverse transcription-PCR (qRT-PCR) documented, in the ATRA-treated group, a strong decrease (about 70%; *P* < 0.05) of *Hsp90* transcripts compared to untreated *Aspergillus* conidia (Fig. 5C). It was previously reported that the expression of the conidiation-specific genes *AbaA* and *WetA* is reduced in the presence of *Hsp90* inhibitor (14). To confirm that fungistatic activity of ATRA is Hsp90 related, we also quantified the expression of *AbaA* and *WetA*; qRT-PCR analysis showed that ATRA-induced and ATRA-reduced conidia formation associates with the inhibition of *Hsp90* expression and decreased *AbaA* (about 60%) and *WetA* (about 60%) transcripts in *Aspergillus* cultures (Fig. 5D). Similar results were also observed at 62 µM ATRA concentration (data not shown). Hsp90 is also reported to interact with the catalytic subunit of calcineurin (15), necessary for correct hyphal growth (16). We investigated ATRA-induced inhibition of the calcineurin pathway and quantified the expression of *CrzA*, a major signaling component of the zinc finger transcription factor. *CrzA* expression was strongly reduced (about 40%) in the presence of ATRA (Fig. 5D). Those data demonstrated that ATRA is a potent inhibitor of fungal *Hsp90* expression and Hsp90-related genes and that its inhibition likely contributes to the fungistatic activity of ATRA against *Aspergillus in vitro*.

### Heat shock protein 90 ATP-binding site is a target of ATRA.

**FIG 6:**
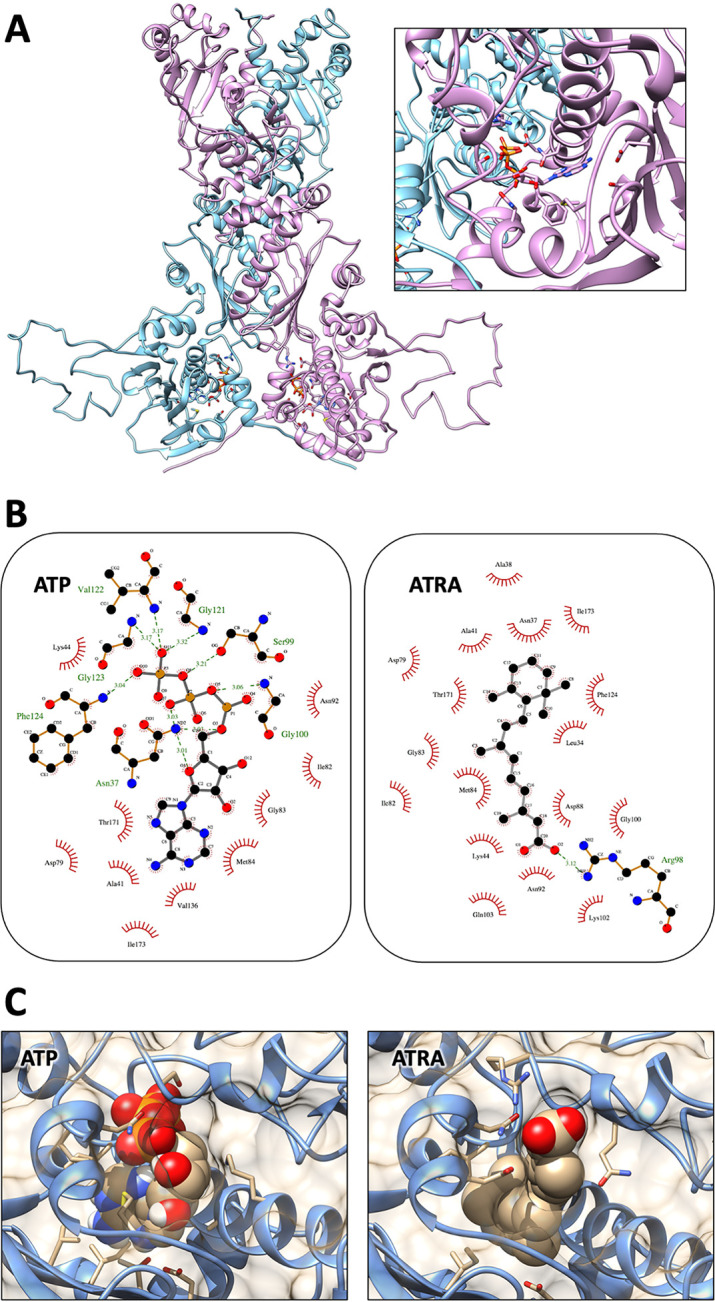
Heat shock protein 90 is a pharmacological target of ATRA. (A) Structural model of Aspergillus fumigatus Hsp90 dimeric structure. Magnification of Aspergillus fumigatus ATP-binding site, including the ATP molecule from the template, colored by atom type (right). (B) Schematic view of the best molecular docking complexes between Hsp90 Aspergillus fumigatus. ATP (left) and ATRA molecule (right). The hydrogen bonds have been indicated with green dashed lines between the interaction partners. The residues composing the active sites that are in proximity of the molecules are shown. Images have been produced using the LigPlot+ software. (C) Best molecular docking complexes between Aspergillus fumigatus Hsp90 with ATP (left) and ATRA molecules (right). The α-helices and loops are shown as light blue spirals and wires, respectively. The molecular surface is shown, and the ligand molecules, hosted in the Hsp90 ATP-binding site, are depicted by sphere representations. Panels A and C have been produced using the Chimera program.

Hsp90 is a chaperone protein required for the activation and stabilization of a wide variety of client proteins, many of them involved in crucial cellular pathways (17). In the absence of A. fumigatus Hsp90 X-ray structure, the crystal structure of Hsp90 from Saccharomyces cerevisiae, with PDB ID 2CG9 (18), whose sequence shares around 78% of identity and 95% of query cover with A. fumigatus, has been used as a template to generate the A. fumigatus Hsp90 model structure (Fig. 6A). A structural bioinformatic analysis was performed to investigate whether Hsp90 may be an ATRA-specific pharmacological target against *Aspergillus* growth. The ATRA structure, as observed with the ATP molecule, completely filled the Hsp90 ATP-binding site in the N-terminal domain of the Aspergillus fumigatus model structure (Fig. 6B and C). In particular, the trimethylcyclohexene moiety was located at the bottom of the ATP-binding site, fully stabilized by hydrophobic contacts with the residues (Fig. 6B and C) (19), while the carboxyl-terminal group was involved in hydrogen bonds with residue Arg98 located at the external border of the ATP-binding site. The interaction energy, evaluated by molecular docking simulation, was around −8.9 kcal/mol, a value only slightly below that obtained using the same procedure with the ATP molecule (−9.7 kcal/mol). These results strongly supported that ATRA molecule may be considered a competitive inhibitor of the Hsp90 ATP-binding site, in particular at high concentrations, and further confirm Hsp90 as a target of ATRA-induced fungistatic activity.

## DISCUSSION

Over the last decades, the incidence of IFIs constantly increased. Moreover, the use of fungicidal drugs in the treatment of fungal diseases led to the development of resistance burden (20). Here, we documented that ATRA displayed a strong fungistatic activity in *Aspergillus* cultures and reduced mortality in a model of IPA in cortisone acetate-immunosuppressed rats. Our preliminary data also showed that the therapeutic benefit of ATRA is dose dependent and, most importantly, the outcome comparable to that obtained with posaconazole, used as the current standard care for the treatment of IPA. Our *in vitro* and *in vivo* findings help to explain some observations previously reported in the clinical practice. In leukemic patients treated with ATRA and other drugs, a lower occurrence of overall fungaemic events has been observed (21, 22). Thus, the low clinical incidence of fungal infections is likely linked to the ability of ATRA to rapidly differentiate leukemic promyelocytes (23) but, more importantly, to its direct antifungal and immunomodulatory properties. In this light, we also documented a synergistic therapeutic activity of suboptimal doses of ATRA combined with the subinhibitory concentration of the antifungal drug amphotericin B against *Aspergillus* conidia germination. This promising interaction could provide an important potential advantage with the use of ATRA in clinical practice, allowing for reduction of the dosage of classic antifungal drugs with a greater control of their side effects as well as a potential better control of the development of fungal drug resistance. However, further experimental *in vivo* studies are required to establish an optimal therapeutic protocol for the use of ATRA in association with antifungal drugs against IPA and systemic *Aspergillus* infections.

It is well-known that conidial germination represents a crucial step in the pathogenesis and progression of IPA. Alveolar macrophages are the first important line of the host’s immune defense against *Aspergillus* conidia. In healthy individuals, lung resident macrophages are able to rapidly engulf and kill inhaled conidia and are not capable of internalizing hyphal cells (24). In immunocompromised patients, nonphagocytosed conidia produce germ tubes that elongate into hyphae in the lung tissue, causing a systemic infection for their capacity to invade blood vessels. Here, we demonstrated that ATRA increases, in a dose-dependent manner, the number of adherent and internalized resting *Aspergillus* conidia by macrophages, suggesting its modulatory action on innate immunity. Our results were in line with previous studies showing that ATRA is able to enhance macrophage phagocytosis against *Candida* yeasts and to favor healing of pneumonia by Pneumocystis carinii in an affected patient (23, 25). ATRA, by suppressing the conidial germination and hyphal growth, could exert a crucial role in facilitating the conidia uptake and their elimination by phagocytic cells, preventing the spread of infection as well as host tissue damage caused by hyphal invasion. Considering the crucial role of macrophages in *Aspergillus* conidia clearance and that the phagocytic activity is impaired in severely immunocompromised patients, the impact of ATRA on phagocytic activity of macrophages is quite relevant. Recent studies also support the hypothesis that efficacy of vitamin A and ATRA alone or in combination with other drugs is related to the capacity to stimulate the monocyte-mediated immune response (26).

Recent works indicate that fungal Hsps are implicated in several processes, including pathogenicity, phase transition, and antifungal drug resistance (27). The function of Hsp90 in *Aspergillus* has been also investigated, and genetic and pharmacologic studies indicate an important role of Hsps in conidiation and in cell wall stress-compensatory mechanisms (27). Here, we report a reduced Hsp90 expression in ATRA-treated *Aspergillus* conidia cultures. Thus, Hsp90 could represent an important target for inhibiting replication and controlling fungal drug resistance. It was demonstrated that Hsp90 itself is subjected to posttranslational modification in a cell cycle-dependent manner. In S. cerevisiae, during the S phase of the cell cycle, the tyrosine kinase Swe1 phosphorylates a residue in the N terminus of Hsp90 that facilitates chaperone interaction with numerous client proteins (28). A role for Hsp90 in Candida albicans cell cycle progression has been also established and a molecular link between Hsp90, cell cycle regulation, and morphogenesis defined, identifying Hsp90 as an important player in cell cycle progression (29). Besides Hsp90, our results showed that ATRA downregulates transcription of other genes, in particular, AbaA and WetA. Their inhibition is reported in association with decreased conidia formation (30). We documented the downregulation of CrzA expression in ATRA-treated *Aspergillus* cultures. CrzA is involved in the calcineurin pathway and hyphal growth (31).

In order to better elucidate how ATRA targets the expression of Hsp90, molecular docking demonstrated that ATRA can fit the Hsp90 ATP-binding site in the N-terminal domain of the Aspergillus fumigatus model structure. Hsp90 is an ATP-dependent molecular chaperone which is essential in eukaryotes. Hsp90 is required for the activation and stabilization of a wide variety of client proteins, and many of them are involved in important cellular pathways (32). Structurally, Hsp90 is a flexible dimeric protein composed of three different domains which adopt structurally distinct conformations. ATP binding triggers directionality in these conformational changes and leads to a more compact state. Posttranslational modifications of Hsp90, such as phosphorylation and acetylation, may provide another level of its regulation. Phosphorylation and acetylation influence the conformational cycle, cochaperone interaction, and interdomain communications (33). Targeting Hsp90 to reduce stress response compensatory pathways with fungal-specific inhibitors of ATRA may represent an attractive novel antifungal strategy (34).

In conclusion, we documented the efficacy of ATRA in a rat model of IPA and its synergistic capacity to improve the efficacy of antifungal agents *in vitro*. ATRA, for its direct fungistatic action targeting the expression of fungal Hsp90, combined with its immunoadjuvant effect against opportunistic fungi, may represent a promising novel candidate in the armamentarium of systemic therapeutic strategies for the treatment and/or prophylaxis of IPA and IFIs, either alone or in association with conventional antifungal drugs.

## MATERIALS AND METHODS

### Microorganism and fungal cultures and treatments.

The Aspergillus fumigatus strain obtained from a clinical isolate, according to routine hospital laboratory procedures, was grown on Sabouraud dextrose agar (SDA) (Difco, Detroit, MI) supplemented with chloramphenicol. Resting conidia were obtained from the SDA-grown fungal culture as previously described (11). For *in vitro* studies, ATRA (catalog no. R2625; Sigma-Aldrich, Milan, Italy) and amphotericin B (Fungizone) (Sigma-Aldrich, Milan, Italy) were tested in the range of 1 to 0.06 mM (300 to 37.5 µg/ml) and in the range of 50 to 0.09 µg/ml, respectively. To investigate the potential synergistic interaction between ATRA and AMB on the conidial germination, resting conidia were plated at the density of 1 × 10^5^ in 96-well microplate in RPMI 1640 with 10% fetal calf serum (FCS; Euroclone, Milan, Italy), supplemented with penicillin (100 U/ml) and streptomycin (100 µg/ml) and treated with different concentrations of ATRA or AmB, alone or in combination. In the combined treatment, ATRA was given 30 min before amphotericin B. Fungal cultures were maintained at 37°C and monitored during the first 30 h after the treatments.

### Evaluation of fungistatic activity.

Aspergillus fumigatus development was followed using an optical microscope (Carl Zeiss, UK) with a ×40 magnification objectives lens. *Aspergillus* conidia vitality was evaluated microscopically by the trypan blue dye exclusion method.

### *In vitro* antifungal susceptibility testing.

The synergistic antifungal activity of ATRA in combination with the antifungals AmB or POS (Sigma-Aldrich) against Aspergillus fumigatus was also assessed using the broth microdilution method as described in M38-A2, a document produced by the Clinical and Laboratory Standards Institute (35). RPMI 1640 2% glucose with l-glutamine (Sigma-Aldrich, St. Louis, MO, USA) buffered to pH 7.0 was used as fungal culture medium. A. fumigatus strain was grown on Sabouraud dextrose agar (Difco Laboratories, Detroit, MI, USA) supplemented with chloramphenicol. Resting conidia were harvested by washing the slant cultures with sterile saline as previously described (11). *Aspergillus* inoculum was set to 0.4 × 10^4^ to 0.5 × 10^5^ CFU/ml, standardized spectrophotometrically. We added 100 µl of this suspension to 96-well flat-bottom plates. Twofold serial dilutions of ATRA (from 150 µg/ml to 37.5 µg/ml), AmB (from 50 µg/ml to 0.09 µg/ml), and POS (4 µg/ml to 0.00005 µg/ml) were prepared in RPMI 1640 medium. *Aspergillus* conidia were treated with 100 µl of each antifungal drug either alone or in combination with ATRA. Positive (100 µl of RPMI medium with 100 µl of conidia) and negative controls (200 µl of RPMI containing ATRA alone or in association with the antifungals without conidia) were included in all experiments. The plates were incubated with agitation at 30°C for 24 h. The MIC was determined spectrophotometrically at 510 nm with an enzyme-linked immunosorbent assay (ELISA) reader. For each compound, the MIC was defined as the lowest drug concentration that produced a significant inhibition (MIC ≥ 50) of fungal growth compared with the drug-free control. The MICs were tested in three independent experiments carried out in triplicate.

### Animals, fungal infection, and treatment.

Sprague-Dawley rats (Envigo, Huntingdon, UK) were immunosuppressed by cortisone 21-acetate (catalog no. C3130; Sigma-Aldrich, St. Louis, USA) treatment subcutaneously administered at 150 mg/kg body weight 7 days before the day of infection; immunosuppression was maintained with 80 mg/kg of cortisone 21-acetate for 3 days a week until the end of the experiment. As the immunosuppression treatment started and throughout the study, rats received a low-protein (8%) diet, as well as drinking water containing tetracycline hydrochloride antibiotic (260 mg/liter; catalog no. T3383; Sigma-Aldrich), supplied *ad libitum*. Rats were then infected with 5 × 10^5^ *Aspergillus* conidia in 0.1 ml of saline solution by oropharyngeal aspiration. For treatment, ATRA (Sigma-Aldrich) was resuspended in 100% dimethyl sulfoxide (DMSO) at 50 mg/ml and stored at −20°C in the dark. Before using, ATRA was diluted in peanut oil (catalog no. P2144; Sigma-Aldrich) in order to obtain a 2-mg/kg/2 ml suspension ready for *in vivo* administration. The posaconazole oral suspension was obtained by diluting the clinical drug Noxafil in water at a concentration of 4 mg/10 ml (Merck Sharp & Dohme, Kenilworth, NJ, USA). Posaconazole treatment started concurrently with the infection (day 0), and it was administered for 5 days a week until the end of the study. As a negative control, the vehicle was prepared by adding 2% DMSO in peanut oil and 5% glucose to water. In the prophylaxis schedule optimization experiment, three groups of rats (*n* = 10) were treated with ATRA by intraperitoneal (i.p.) injection for 7 days before infection (day 0) and then for 5 days a week up to the end of the study.

The mortality rate was recorded daily until the end of the studies. All animal procedures were carried out in accordance with the standards established by the Animal Ethical Committee of Takis.

### Phagocytosis assay.

For phagocytosis assay (36), human macrophages U-937 (1 × 10^6^ cells/ml) were incubated overnight in 6-ml polypropylene tubes in Dulbecco’s modified Eagle’s medium (DMEM; Gibco, Milan, Italy) supplemented with 2% (vol/vol) FCS, 2 mM l-glutamine, 100 IU/ml penicillin, and 100 µg/ml of streptomycin with vehicle (DMSO) or different ATRA concentrations (1, 5, and 10 μM). After overnight incubation, the cells were incubated for an additional 1 h at 37°C with unopsonized resting *Aspergillus* conidia at an effector-to-fungal cells ratio of 1:10 before being evaluated for internalization and visualized by light microscopy. Phagocytic cells were separated from nonphagocytosed A. fumigatus by centrifugation on a fetal serum gradient. A 0.1-ml sample of the harvested phagocytic cells was used for cytospin preparation. After Diff-Quik staining, fungal cell internalization was expressed according to the following formula: percentage of internalization = number of cells containing one or more fungal cells/100 cells counted. In parallel, macrophage phagocytosis was also performed in 24-well plates for a real-time analysis of phagocytosis by microscopy. U-937 macrophages cultured at 1 × 10^6^ cells/ml were treated overnight with ATRA and incubated for an additional 1 h at 37°C with *Aspergillus* conidia as described above.

### Western blot analysis.

For Western blot analysis, after extraction, 20 μg of total proteins was blotted onto nitrocellulose membranes and incubated with rabbit anti-Hsp90 (1:1,000; Cell Signaling Technologies, Danvers, MA, USA) and rabbit anti-glyceraldehyde-3-phosphate dehydrogenase (GAPDH) (1:20,000; Gentex, Zeeland, MI, USA), followed by incubation with horseradish peroxidase (HRP)-conjugated anti-rabbit IgG (1:2,500; Santa Cruz Biotechnology, Dallas, TX, USA). Revelation and densitometric blots were performed in three different experiments.

### RNA extraction and real-time reverse transcription-PCR.

A sample of frozen biomass was placed into a 2-ml extraction tube containing beads and disrupted using TissueLyser. RNA was isolated using TRI reagent (Sigma-Aldrich) according to the manufacturer’s instructions. To verify the general purity of the RNA, a NanoDrop instrument was used. One microgram of total RNA was reverse transcribed by a QuantiTect reverse transcription kit (Qiagen, Hilden, Germany) following the manufacturer’s instructions. qRT-PCR analyses were performed using 20 ng cDNA per well in triplicate with the SYBR Green master mix (Applied Biosystems, Foster City, CA USA) (37, 38) according to the manufacturer’s instructions. Reactions were run on 7900HT system (Applied Biosystems).

### Heat shock protein 90 simulation methods.

Protein-ligand molecular docking has been used to evaluate binding mode and energy between A. fumigatus Hsp90, ATRA [2E,4E,6E,8E)-3,7-dimethyl-9-(2,6,6-trimethylcyclohexen-1-yl) nona-2,4,6,8-tetraenoic acid] and ATP, its natural cofactor. The docking simulations have been executed using the AutoDock Vina 1.1.2 program through the AutoDock Vina PyMOL plugin (https://www3.mpibpc.mpg.de/groups/de_groot/dseelig/adplugin.html) (PyMOL molecular graphics system version 1.5.0.4; Schrödinger, LLC) (39). The ATRA and ATP structural files (sdf files) have been obtained from the PubChem compound database (https://pubchem.ncbi.nlm.nih.gov) and have been converted into mol2 files and filled with hydrogens using the Open Babel program (https://openbabel.org). The crystal structure of Hsp90 from S. cerevisiae has been used as a template to generate the A. fumigatus Hsp90 model structure. The model has been generated using the SWISS-MODEL protein modeling tool (https://www.swissmodel.expasy.org) (40). After a structural check, the model has been used as a receptor for the molecular docking simulations of ATP and ATRA in the ATP-binding site. Thirteen side chains (Leu34, Asn37, Lys44, Asp79, Ile82, Met84, Asp88, Lys102, Gln103, Phe124, Val136, Thr171, and Ile173) belonging to the Hsp90 ATP-binding site have been considered rotatable to improve the conformational search during the docking simulations.

### Statistical analysis.

For *in vitro* experiments, statistical analysis was performed using an unpaired two-tailed *t* test between control and experimental groups. For *in vivo* studies, survival curves were determined by the Kaplan-Meier method, and the log-rank test was used to conduct comparisons between the groups. Test of statistical significance was two-sided; a value of *P < *0.05 was considered to be statistically significant.
